# Implanted Radio Telemetry in Orangutan Reintroduction and Post-release Monitoring and its Application in Other Ape Species

**DOI:** 10.3389/fvets.2019.00111

**Published:** 2019-04-16

**Authors:** James G. Robins, Simon Husson, Agus Fahroni, Ian Singleton, Matthew G. Nowak, Gerhard Fluch, Karmele Llano Sanchez, Andhani Widya, Peter Pratje, Marc Ancrenaz, Nigel Hicks, Benoit Goossens, Thierry Petit, Rashid Saburi, Chris Walzer

**Affiliations:** ^1^Orangutan Appeal UK, Effingham, United Kingdom; ^2^Department of Behavioural Biology, Universität Wien, Vienna, Austria; ^3^Research Institute of Wildlife Ecology, University of Veterinary Medicine, Vienna, Austria; ^4^Borneo Orangutan Survival Foundation, Bogor, Indonesia; ^5^Sumatran Orangutan Conservation Programme, Medan, Indonesia; ^6^Department of Anthropology, Southern Illinois University, Carbondale, IL, United States; ^7^Yayasan International Animal Rescue, Ketapang, Indonesia; ^8^Frankfurt Zoological Society, Jambi, Indonesia; ^9^HUTAN, Kinabatangan Orangutan Conservation Project, Sandakan, Malaysia; ^10^Orangutan Veterinary Aid, Launceston, United Kingdom; ^11^Organisms and Environment Division, Cardiff School of Biosciences, Cardiff University, Cardiff, United Kingdom; ^12^Sabah Wildlife Department, Danau Girang Field Centre, Kota Kinabalu, Malaysia; ^13^Sabah Wildlife Department, Kota Kinabalu, Malaysia; ^14^Sustainable Places Research Institute, Cardiff University, Cardiff, United Kingdom; ^15^DVM, Zoo de La Palmyre, Les Mathes, France; ^16^Wildlife Conservation Society, New York, NY, United States

**Keywords:** great apes, technology, orangutan, post-release monitoring, rehabilitation

## Abstract

Designed as a new method to facilitate the reintroduction and post-release monitoring of orangutans and other apes, implanted radio-telemetry (IRT) was developed and first deployed in 2009. Since that time, it has been necessary to collate and review information on its uptake and general efficacy to inform its ongoing development and that of other emerging tracking technologies. We present here technical specifications and the surgical procedure used to implant miniaturized radio transmitters, as well as a formal testing procedure for measuring detectable transmission distances of implanted devices. Feedback from IRT practitioners (veterinarians and field managers) was gathered through questionnaires and is also presented. To date, IRT has been used in at least 250 individual animals (mainly orangutans) from four species of ape in both Asia and Africa. Median surgical and wound healing times were 30 min and 15 days, respectively, with implants needing to be removed on at least 36 separate occasions. Confirmed failures within the first year of operation were 18.1%, while longer distances were reported from positions of higher elevation relative to the focal animal. IRT has been a transformational technology in facilitating the relocation of apes after their release, resulting in much larger amounts of post-release data collection than ever before. It is crucial however, that implant casings are strengthened to prevent the requirement for recapture and removal surgeries, especially for gradually adapting apes. As with all emerging technological solutions, IRT carries with it inherent risk, especially so due to the requirement for subcutaneous implantation. These risks must, however, be balanced with the realities of releasing an animal with no means of relocation, as has historically been, and is still, the case with orangutans and gorillas.

## Introduction

Most ape species are subject to population pressure across their range due to diminishing habitat quality and/or human wildlife conflict and hunting (1–7). As a direct result of these threats, the Javan gibbon (*Hylobates moloch*) is Endangered, while the three species of orangutan (Sumatran: *Pongo abelii* and *P. tapanuliensis*; Bornean: *Pongo pygmaeus*) and the western lowland gorilla (*Gorilla gorilla gorilla)* have become critically so (8). Habitat loss, poaching and displacement has also led to increasing numbers of displaced apes, as well as the proliferation of rehabilitation facilities in Africa and Asia. These facilities typically operate with the goal of managing otherwise healthy orphaned animals through pre-release training and reintroduction programmes.

In the context of species conservation, reintroduction is the only logical step that rehabilitation centers should be making. Yet the practice carries with it welfare and disease risks for both released and resident wild animals (9–11). Post release monitoring is the most effective method of assessing how to reduce these potential risks, as well as providing the means to better understand the process of adaptation, assessing the suitability of a given pre-release rehabilitation protocol and release site, and to formulate criteria for assessing reintroduction success. Despite this, its application among several species has been limited (12). Post-release monitoring is severely restricted by an inability to relocate animals regularly, as is often the case for wide-ranging apes which may also show limited social interactions or vocalizations, e.g., orangutans. Reintroduction successes and failures may thus remain unknown for the vast majority of animals.

Radio telemetry has the capacity to transform our ability to conduct adequate monitoring and data collection, through the development of methods specifically designed to locate individuals after release. Among its key benefits are the unequivocal identification of individuals and the facilitation of data collection (13), and the ability it conveys to reintroduction specialists to intervene to promote welfare or prevent potential conflict situations involving released animals. Its biggest impediment among apes, however, has been the absence of appropriate species-specific attachment systems for these dexterous, intelligent, and strong animals (14–16). Decisions not to employ available tracking devices may also be influenced in some cases by their prohibitive cost per individual released, weight, and the historically poor fix rates of commonly used GPS devices because of canopy closure and topographic conditions (17, 18). While radio collars have proven successful in monitoring prosimians [*Galago alleni:* (19); *Galago senegalensis*: (20)], some monkeys [*Ateles geoffroyi:* (21, 22); *Aotus azarai:* (23)], and reintroduced chimpanzees [*Pan troglodytes:* (24–26)] it has not been possible to fit them on orangutans because of their small heads, relatively wide necks and soft throat pouches (27).

In response to these issues, the Research Institute of Wildlife Ecology in Vienna developed new subcutaneous radio telemetry transmitters and a corresponding surgical implantation method in 2009. Since then, implanted radio telemetry (IRT) has been adopted by numerous ape reintroduction projects such that the collation of information on its application is now necessary to inform its continued development and use in facilitating post release monitoring. This is particularly necessary given that tracking technologies are constantly evolving and improving (28). Here we describe (1) the technical specifications of equipment used; (2) the surgical implantation method; and through surveys with end users we also review (3) general device uptake; (4) observed distances and ranges of implants; and (5) practitioner perceptions and recommendations. In our discussion, we also identify the key issues and challenges associated with implanted devices and we compare these with more established applications of radio telemetry. The scope of this technology is potentially vast across many different genera so we intend not only to bring IRT to the wider attention of biologists but also to assist all those interested in further developing wildlife tracking technologies.

## Materials and Methods

### Miniaturized Radio-Telemetry Transmitters—Technical Specifications

**Figure 1:**
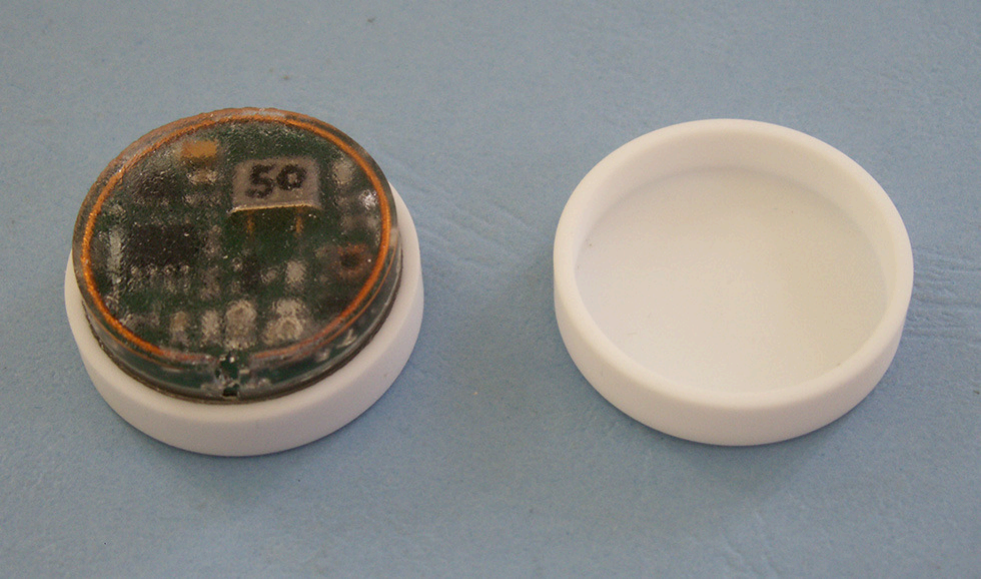
An open transmitter showing its circuitry and ceramic casing.

To be suitable for sub-cutaneous implantation each device comprises a miniaturized circuit board, battery and VHF transmitter housed in inert and secure ceramic circular casings to protect against liquid ingress (Figure 1). Two small circular VHF transmitter implants have been developed with different battery options: a smaller iteration with a 280 mAh battery (*d* = 28 mm, *h* = 10 mm, 14 g), and a larger iteration with a 540 mAh battery (*d* = 28 mm, *h* = 12 mm, 17 g). Based on diameter alone, this makes both implants marginally smaller than a United States 50 cent coin. Since 2009, 481 transmitters were sold up to the end of December 2016, most of which carried the larger battery (362) vs. the smaller (119). Each device emits a pulse of 0.01 s duration at 1.5 s intervals. The electronic circuit is housed in a computer numerically controlled (CNC) engineered, inert ceramic casing, hermetically sealed with specially formulated epoxy glue.

The on/off timing schedule of each transmitter is pre-programmed during production in response to how many hours per day the research team requires transmission. The start time of this schedule is determined by the user via magnetic switch. The unit is controlled by a very low-power-timer-circuit that allows for a life span of several years. Shorter daily transmission periods result in longer battery lifespan; an 8-h daily transmission schedule provides an estimated battery life of 33.6 months. Various frequency bands are available. To track the signal of the implants, a standard tracking receiver and adequate directional antenna covering the frequency range is required.

### Implantation Surgery and Recovery

Following induction of surgical anesthesia transmitters are implanted in a surgically created subcutaneous pouch in the cranio-dorsal cervical area. After hair-trimming and standard aseptic pre-operative surgical preparation of the skin, a 4cm paramedian incision is made ~4 cm distal to the base of the occipital bone. This is followed by blunt dissection of the subcutaneous tissues caudal and lateral to the initial incision to facilitate subcutaneous dorsal parasagittal-midline insertion of the transmitter. Subcutaneous tissues are sutured to secure the transmitter before the skin is closed with absorbable monofilament suture material using an interrupted intra-dermal suture pattern. The transmitter is implanted specifically with the plane of the transmission face facing the suture line to maximize detection efficacy. Treated animals are then maintained in smaller enclosures until the wound is fully healed. Wound healing times are subject specific, but can reasonably be expected to complete within ~2 weeks post-surgery. The healing process is regularly evaluated by veterinarians and any healing abnormalities are treated accordingly. Placement of the transmitter lateral to the spinous processes in this position reduces the likelihood of traumatic damage post operatively.

### Questionnaire and Device Outcome Table for End Users

A questionnaire and a device outcome table were designed for primate reintroduction practitioners who have used IRT with non-human primates to assess general use and effectiveness. These documents were emailed to practitioners between July 2013 and March 2017, with the nature and purpose of the questionnaire clearly explained. Consent to participate was implied upon completion of the questionnaire. The device outcome table provided quantitative biodata on animals implanted, surgery and recovery times, implant battery lives and outcomes. The questionnaire provided data on the current perceptions of IRT from field practitioners. Questions were mostly close-ended multiple choice to facilitate completion and quantitative analysis, although some sections required descriptive detail. Due to the relatively small number of projects that are using this technology, descriptive statistics and frequencies were used in analyses of the questionnaires.

### Distance Testing Protocol

To give an indication of the signal range capability of implanted transmitters, instructions were given to four field projects to measure the angle through which audible transmitter signals could be detected at increasing 50 m intervals from the focal animal while it remained stationary (typically while resting, foraging, or in an acclimatization enclosure). Participants were asked to conduct this signal test under three conditions where possible: (1) at similar elevations to those of the focal animal; (2) from downhill positions; and (3) from uphill positions. Data were recorded for 13 different implanted animals until the point at which each signal became undetectable. Each person conducting the tests was asked to have their earphones plugged in with maximum gain to increase signal detectability. All tests were conducted in clear weather. Each 50 m interval, as well as the elevation of subject animals and of the person conducting the test were measured by hand held GPS units (Garmin CSX).

For each 50 m testing interval all differences in elevation between the animal and the receiver were pooled and categorized, with values ≥300 m forming one group due to diminishing sample sizes. Two independent groups for comparison were then created to test the following three hypotheses: (1) either side of the median elevation differential: when values were split down the middle would either group produce significantly better signal range compared to the other? (2) either side of the mean elevation differential: would a more pronounced division of samples which isolated the largest differences in elevation produce a significant difference in audible signal range? (3) positive vs. negative positions: does being either uphill or downhill from the animal result in better signal range? Non-parametric Mann Whitney *U* analysis was conducted on each of these three comparative groups due to non-normal data distribution. A simple linear regression was also conducted across the entire sample to predict the effect of increasing distance on audible signal range. Statistical analyses were conducted in IBM SPSS Statistics 23.

### Distance Testing Site Descriptions

Distance testing data were collated from four ape reintroduction projects; two with Bornean orangutans *Pongo pygmaeus* (Tabin Wildlife Reserve, Sabah, Malaysia & Bukit Batikap Protection Forest, Central Kalimantan, Indonesia) one with Sumatran orangutans *Pongo abelii* (Jantho Pine Forest Nature Reserve, Aceh, Indonesia) and one with Javan gibbons *Hylobates moloch* (Gunung Tilu Nature Reserve). Each is an undulating, hilly rainforest at low elevation <500 m asl, except for Gunung Tilu which is montane forest at 1,300–1,800 m.

### Ethics Statement

Procedures in Malaysia, Indonesia, Gabon and the Congo were carried out according to the requirements of national animal welfare and animal use legislation by registered and qualified veterinarians in registered institutions, IUCN Guidelines for Reintroductions and Other Conservation Translocations. No additional permits were required.

The main issue is that in most of the countries we work, there are no specific national animal welfare and animal use legislation beyond the permit to work on the animals and this is covered by the stringent permits of the registered institutions [Indonesia, Gabon, Congo] or government institutions [Malaysia].

## Results

### Device Outcome Table Results

#### Response Rate and Species Represented

A total of 11 different ape release projects were identified by the first author as having adopted IRT. We received nine fully completed questionnaires representing the views of all but one of the groups that have historically employed IRT; one organization ran multiple projects. The device outcome tables were returned by respective projects in varying degrees of completion. Two species of orangutan (*Pongo pygmaeus* and *Pongo abelii*); western lowland gorilla (*Gorilla gorilla gorilla*), and the Javan gibbon (*Hylobates moloch*) are represented in our dataset, and, at the time of writing, remain the only species of primates in which IRT has been investigated in a field setting.

#### Biodata and Uptake

Since their initial development in 2009 until March 2017, a minimum total of 291 surgeries have resulted in transmitters being implanted into 256 individual apes. The variation in weight of focal animals ranged from 5.2 to 170 kg. For the lightest ape implanted in our dataset, the larger implant therefore represents 0.33% of the animal's total body weight. Additional biodata of implanted animals are presented in Table 1.

**Table 1:** Biodata of implanted apes per species.

**Species name**	**Surgeries**			**Age @ first implantation (yrs)**				**Bodyweight at implantation (kg)**		**Date range**	**Project locations**
	**#**	**% ♂**	**% ♀**	***N***	**Mean**	**SD**	**Range**	***N***	**Range**		
*Pongo pygmaeus*	206	37.9	62.1	173	13.3	5.1	5–25	161	11–91	2009 - ongoing	Sabah, Malaysia; Kalimantan, Indonesia
*Pongo abelii*	80	51.2	48.8	75	9.1	4.1	5–22	70	11.5–62	2010 - ongoing	Sumatra, Indonesia
*Gorilla gorilla gorilla*	3	33.3	66.6	3	17	9.8	6–25	3	50–170	2013–14	Gabon; Republic of the Congo
*Hylobates moloch*	2	50	50	2	14.5	2.5	12–17	2	5.2–6	2015	West Java, Indonesia

*% ♂, percentage of males implanted; % ♀, percentage of females implanted; SD, standard deviation from the mean*.

#### Implantation Surgeries and Healing

From the data available detailing surgical implantation procedures (*n* = 155), as measured from the first incision to closing of the wound, over three quarters (78%) were completed under 45 min with a mean of 26 ± 8.4 min. The median duration across the entire group was 30 min, with a range of 5–88. Post-surgical healing durations (*n* = 169), as measured in days until the wound was deemed entirely healed by project veterinarians, had a wide documented range of 3–127 days, but a relatively low median value of 15 days, within which more than half of animals (57.4%) had fully healed by primary intention after initial stitching. In a small number of cases practitioners reported wounds opening in the days and weeks after surgery. This process was in some cases caused or exacerbated by a few anecdotal reports of persistent interference with wounds and stitches by orangutans, especially so by more feral individuals that had spent less time in rehabilitation facilities. In these cases where the wound edges were no longer held together, wounds were re-sutured or left to heal by secondary intention i.e., granulation tissue matrix filling the wound defect, therefore rendering them more susceptible to complications (infections, seroma) in the healing process.

There were an additional 26 surgeries where implants were removed and immediately replaced with new devices during the same procedure. Predictably, the surgical times (*n* = 9; median = 45; range: 30–120) and healing durations (*n* = 14; median = 32; range: 7–45) typically lasted longer compared with the implantation procedures above, due to the additional work involved during surgery and increased trauma to the soft tissue around the implantation site, respectively.

#### Implant Removals

Transmitters were removed from focal animals on 36 separate occasions. Details of the seven most complex clinical cases, as reported by the respective project leaders and veterinarians, are presented in Table 2. Additionally, seven implants prematurely failed and were found with cracks in their ceramic casing at the time of removal. Implant developers identified that there had been a faulty batch of devices produced with some hairline fractures in their ceramic casings, so a further five transmitters were removed and replaced as a precautionary measure.

**Table 2:** Implanted transmitter removals.

**Species and focal animal biodata**	**Reason for removal, implant status and additional comments**
1) *Pongo pygmaeus* 9yo female	Eight weeks post-surgery the orangutan received multiple bite wounds by a conspecific at implant site while awaiting release. She was retained in clinic for observation and treatment but within 1 week the wound started to dehisce and a fragmented ceramic shard became visible grossly. Implant was fractured with its seal broken. Nine small fragments were retrieved along with the main part of the transponder. The surgical site was grossly contaminated with discharge, necrotic tissue and a dark material believed to be battery contents. There was considerable localized irritant reaction together with secondary infection and tissue necrosis. Another implant was placed 6 months later adjacent to the original surgical site—there was minimal residual fibrosis and surgery was uneventful, healing by first intention within 5 days.
2) *Pongo pygmaeus* 8yo male	After the orangutan had been free roaming in the pre-release forest school, he was brought back to a cage in preparation for release. It was then noticed that the transponder was not transmitting. Efforts were made to reactivate the transponder with several different magnets but with no success. When removing the transponder, it was found that the device had cracked into pieces and within the surgical wound some necrotic tissue was found, possibly due to battery leakage. A replacement was fitted on the same day as the faulty implant was removed, with healing time taking longer than average at 41 days.
3) *Pongo pygmaeus* 7yo female	The orangutan removed the surgical stiches and the surgical wound had to be re-sutured 2 weeks after the initial procedure. Sutures were again pulled out by the animal. The wound could not heal by first intention healing and it was infected, so it was decided to remove the implant almost 4 weeks later to allow the wound to heal by second intention. The implant was not replaced.
4) *Pongo pygmaeus* 5yo male	This was a wild young orangutan therefore it was difficult to check his wound after the 49-min implantation surgery. Six days after surgery an infection was spotted so the orangutan was sedated to clean the wound and remove the implant. During the procedure, the orangutan had a cardio-respiratory arrest and died.
5) *Pongo pygmaeus* 8yo male	Months after successful implantation, the orangutan was seen falling out of a tree. Two days later a heavy branch fell across its shoulders, after which the device became inactive. A small crack in the ceramic casing was visible before it splintered completely under the pressure of pincers during removal. The implant was replaced 14 months later.
6) *Pongo abelii* 6yo male	Orangutan was engaged in some rough and tumble play with a conspecific less than 1 month after the device was fitted when the transmitter stopped functioning. Upon removal, the implant was found to have fractured into several different pieces and there was a severe localized reaction and infection at the transmitter site. Device was not replaced.
7) *Pongo abelii* 9yo male	Animal was very active during recovery, banging its neck and back against the cage such that the wound required re-stitching four times. The skin surrounding the implant site had lacerations and the transmitter protruded about 5 mm. A day later the station manager found parts of the fragmented transmitter on the cage floor, with the animal playing with and sucking other fragments. We suspect the OU took the transmitter from the lacerated skin and bit it. Two fragments of transmitter casing were found but some other parts (including the battery) were not located. The wound was opened and cleaned before another implant was placed a day later.

#### Implant Status and Confirmed Outcomes

**Figure 2:**
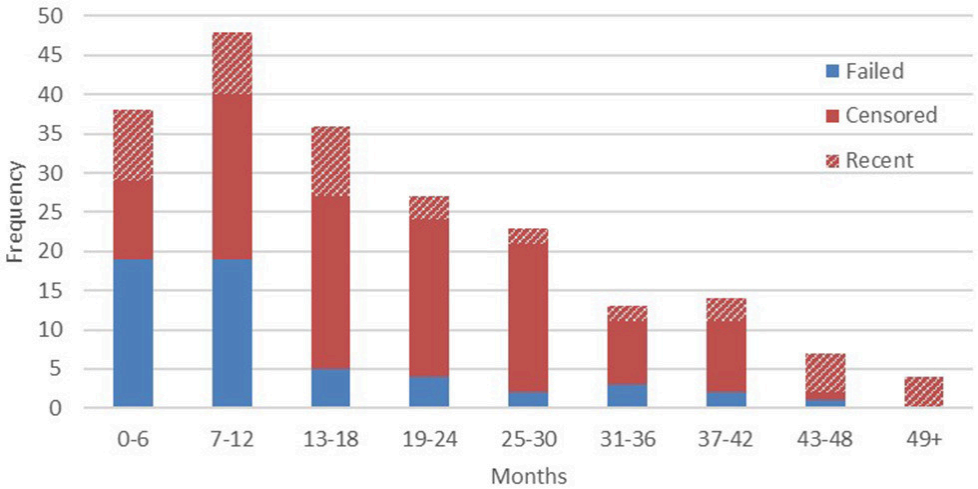
Confirmed vs. censored device outcomes per 6 month interval.

At the time of writing, device life cycle data were available from 210 transmitters. Within that figure, audible signals were detected in the 6 months immediately preceding the latest field update in 21.4% of cases (*n* = 45). Failed devices were confirmed by sightings of the animal without signal being present. The right censoring that we employed, therefore, reflects a high probability that in the majority of cases the remaining outcomes can be considered final, with animals either dispersing, dying, or monitoring effort ceasing beyond a certain point (full results presented below in Figure 2).

Confirmed implant failures decreased as a proportion of total device outcomes with each successive half year period, as the proportion of censored outcomes correspondingly increased. This is most likely due to the majority of animals being monitored intensively only in their first several months after release, until resources are focused on other, more recently released, apes. We should therefore acknowledge here that the high failure rate reported from year one final outcomes, when regular monitoring is more common, would indicate that additional transmitter failures likely go undetected among the censored population, especially given the wide ranging habits of exploratory orangutans. Conversely, our data demonstrate that in a small number of cases (11.9%, *n* = 25) implants also function beyond the end of their expected device lifetime (33.6 months on a typical 8 h transmission schedule), so similarly long transmissions may also go undetected. To date, the longest reported transmission was recorded 57 months after implantation. The animals represented in years four and five post-release are likely individuals who have settled within relatively stable home ranges close to the research base, thus enabling regular signal detection within the typical range of IRT.

### Questionnaire Responses

#### Reliance on IRT

Practitioners were asked “*how many days per month do you sight each released individual?*” The most common response given was 0–3 days per month (*n* = 5), representing 56% of respondents. When asked to highlight the factors responsible for limiting direct observational data collection, the two most common responses given (both *n* = 7) were “*topography of release site”* and “*limited maximum distance and range transmission of implants.”* When asked if they felt able to record behavioral data at the same intensity without implants eight out of nine respondents answered “no”.

#### Managing Faulty/Failed Implants

When asked if they would be concerned with leaving a faulty device within the body of an animal, 56% of questionnaire respondents answered yes. Of these responses, 80% stated explicitly the potential for faulty implants to cause injury to the host animal (i.e., battery leaks and splinters because of cracked casings, potential to migrate within the host organism). In cases where transmitters are known to have failed prematurely, respondents were also asked if they would consider retrieving and replacing them if the animal had already been wild released. Just over half of respondents (56%) said they would attempt to retrieve the faulty implants, with the single most common reason cited being “*signs of damage to the implant,”* although it was also noted by multiple respondents that this would be contingent on their ability to relocate and recapture these animals. For those that said they would not retrieve faulty implants, the most common reason cited was concern about clinical/surgical risks (e.g., anesthesia/darting etc.).

#### IRT Reuse and Practitioner Perceptions

One hundred percent of questionnaire respondents said they would use IRT again. Respondents were also asked to describe their general thoughts on IRT, the biggest issues they have faced when using this technology in the field, and how they would like to see the implants developed in the future. Results are presented in Appendix A1.

### Distance and Range Testing of Subcutaneous Implants

**Figure 3:**
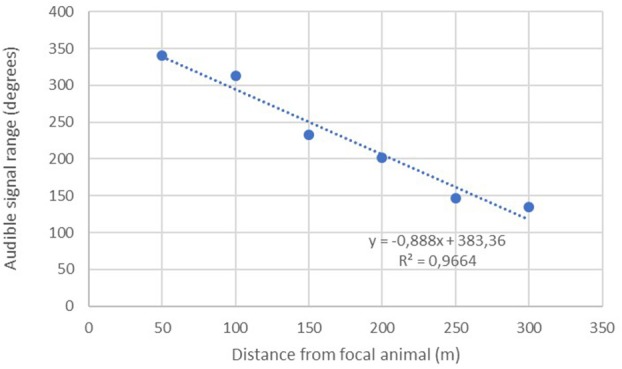
Mean audible signal range measured at increasing 50 m intervals away from focal animals.

A linear regression was conducted to assess the extent to which increasing distance predicted the remaining audible signal range of the implanted transmitters (*n* = 26). Results show a significant inverse relationship with audible range typically decreasing by about 44.39 degrees with each additional 50 m interval traveled away from the focal animal (Figure 3).

**Figure 4:**
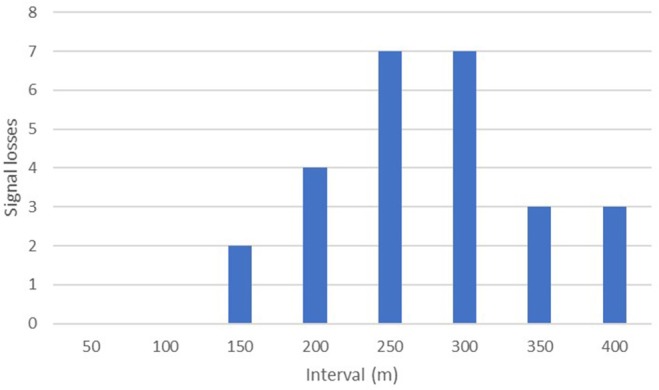
Signal drop outs plotted at their final distance testing interval.

The maximum distance testing interval reached during our formal tests was 400 m; a figure obtained by just three separate implants. However, anecdotal reports from several projects suggest much greater distances can be obtained in special circumstances i.e., when there is little landmass between focal animal and the receiver. Seventy-seven percent (40/52) of our tests up until the 100 m distance interval yielded audible signal ranges in all directions throughout 360°. No signals were lost across the entire sample until we moved past the 150-m interval, with the majority of signal drop outs (14/26) occurring between 250 and 350 m (Figure 4).

When the sample was divided by the median value for each interval into two independent groups consisting of <median (*n* = 67, mean rank = 72.19) and ≥median (*n* = 77, mean rank = 72.77) no significant differences were found: *u* = 2558.500, *z* = −0.086, *p* = 0.931. Similarly, the more pronounced division of samples either side of the mean value for each interval (<mean: *n* = 92, mean rank = 74.92; and ≥mean: *n* = 52, mean rank = 68.21) resulted in no difference: *u* = 2169.000, *z* = −0.950, *p* = 0.342. However, when the test was applied to evaluate whether positive values i.e., being uphill from the focal animal (*n* = 96, mean rank = 77.50) would yield stronger signals compared with negative values (Table 3) i.e., being downhill from the focal animal (*n* = 48, mean rank = 62.50) we found a significant difference across the entire sample: *u* = 1824.000, *z* = −2.084, *p* = 0.037. Our tests show that signals are therefore stronger when the receiver is uphill from the focal animal.

**Table 3:** Uphill vs. downhill range and signal loss characteristics relative to the position of the focal ape, per distance testing interval.

	**Level/uphill receiving position**					**Downhill receiving position**				
	***N***	**Mean signal range and SD**	**Elevation differential**	**Drop outs**	**Drop out differential**	***N***	**Mean signal range and SD**	**Elevation differential**	**Drop outs**	**Drop out differential**
50 m	20	335.4 ± 77.2	7.5	0	–	6	354.8 ± 9	−11.7	0	–
100 m	19	312.7 ± 100.8	10.6	0	–	7	311.9 ± 89.2	−23.9	0	–
150 m	17	263.4 ± 105.3	17.1	2	21.5	9	175.1 ± 99.9	−24.0	0	–
200 m	15	214.9 ± 123.5	23.2	3	40.7	9	178.4 ± 94.1	−28.0	1	−55.0
250 m	11	136.8 ± 85.9	24.6	2	37.0	9	157.8 ± 41.7	−28.2	5	−24.4
300 m+	14	141.6 ± 117.4	22.6	9	23.6	8	123.8 ± 81.6	−26.3	4	−38.8

## Discussion

### General Implications of IRT

The use of IRT, particularly with orangutans, has been transformational as prior to its development, post-release monitoring entirely depended on enough animals remaining within a given release site for long enough to enable reintroduction outcomes to be known (29–31). Researchers had no methods available to improve individual location in the field and there was virtually no information on reintroduction outcomes as a result (16). Over the coming years we can expect that the true value of IRT will be shown through greater data collection and the dissemination of post release outcomes that should guide reintroduction practitioners in adopting more successful release strategies. By facilitating focal follows, IRT has also enabled interventions that have saved the lives of struggling animals thus improving welfare for those individuals greatly. When functioning correctly, the long device lifespan of almost 3 years negates the need for disruptive re-captures of gradually adjusting rehabilitants to replace batteries, re-adjust attachments, or to retrieve transmitters, as seen among other species (32–34).

Having an implant, however, is just one factor involved in locating apes after their release. Release site location and its topography, monitoring effort and the ratio of research assistants to animals released, the number of animals awaiting release, and project financing all dictate the relative difficulty with which released apes can be relocated. Despite the widespread uptake of IRT many animals are still lost or disperse relatively early into their release, as demonstrated in our analysis of device outcomes. Variation in the number of animals released between projects, in particular, means that it is certainly easier for smaller projects to directly observe each animal on a regular basis compared with those conducting large group releases into the hundreds of animals. So, while carrying an implant doesn't necessarily result in the regular observation of *all* animals, this technology has nonetheless greatly advanced the field of ape reintroduction by dramatically increasing the number of animals that theoretically *could* be relocated.

### Comparisons With External Application of Radio Telemetry

A common recommendation found in the literature is that a tracking device should aim to be no more than 5% of an animal's bodyweight (35, 36). At around 0.3% of total bodyweight for the smallest ape in our dataset, the size and weight of implanted transmitters most likely result in negligible effects on locomotive patterns, general behavior, and body condition. There may thus be substantial scope for increased use among a wider range of smaller species, including those outside the primate order. Heavier external devices that exert their weight on just one limb are probably more likely to affect an animal's activity patterns, as demonstrated by relatively small differences in radio collar weight interfering in the grazing behavior of zebras (37), and the mortality of migratory caribou (38). While the positional behavior of most species must be carefully considered before a tracking device is employed, therefore, IRT in apes is largely free from this requirement.

Our distance testing protocol present for the first time a method for the systematic testing of radio telemetry applications. At around 250–350 m, the modal maximum distances at which implanted signals are detected in this study are broadly similar to previous telemetry incarnations for locating galagos *Galago alleni* (19). Anecdotal reports from field teams would suggest that despite the relatively short distances we found during formal testing, signals are also regularly detected from long distance, although typically under rare topographical conditions such as within relatively open basins or across gullies with few central hills or vegetation to block signals. That we found stronger signals from elevated positions relative to the focal animal is consistent with previous research (39), and has important implications for release site choice and design, such as the identification of telemetry “sweet spots” including elevated ridges and trails, as recommended by the IUCN (10).

Aerial signal detection was not possible within this study but would most certainly enable considerably longer detection ranges for VHF-GPS implants (40). Flying unmanned fixed wing drones high above canopy level in grids would enable huge areas of land to be covered and for areas with strongest signals to be identified. This alone would represent a huge advance in post release monitoring by helping projects to more adequately assess the movements of a larger proportion of animals, particularly so for wide ranging species. This may also lead to well performing apes being almost entirely monitored remotely. For now, though, it is important to note that both staff training in good telemetry techniques and employing implant-frequency-specific antennas are essential when working with such low-output VHF transmitters.

To date, the requirement for sub-cutaneous implantation has severely constrained device functionality by limiting the maximum size of transmitters and their components. These are low output VHF capable devices, without store on-board data logging, accelerometers, GPS receivers, RFID sensing, satellite data retrieval, nor remote tracking capabilities. Their functionality thus falls dramatically short of most off-the-shelf collars produced by established wildlife telemetry companies that typically allow end users to either remotely track animal movements through two-way satellite communication, or to download stored positional data in the field within a certain range of the focal animal. With several large release programmes unable to directly observe individual animals on a regular basis due to a lack of resources or changing research priorities, there is a clear need to incorporate more sophisticated data logging and remote monitoring methods, as highlighted by end users in our survey. There is hope that the International Cooperation for Animal Research using Space (ICARUS; http://icarusinitiative.org) may provide the necessary data-download technology for similar small GPS implants in the near future (41). Spatial analysis provided by GPS capable devices would also provide improved mortality data, as currently IRT can only facilitate the homing of animals that stay within the relatively short range of its VHF transmitters.

### IRT Faults and Risks

Perhaps the greatest drawback of IRT is that animals must undergo anesthesia and surgery to place the device along with a post-operative recovery period to monitor wound healing. Several cases of self-inflicted trauma to surgical wounds, especially by wild translocated orangutans and otherwise more feral individuals resisting treatment may explain the wide variation in healing between some individuals. Other confounding factors, including surgical technique, suture materials used, and post-operative veterinary care provided may also explain the wide variation we present in healing times. During the initial years of these implantation efforts regular training workshops were carried out to guarantee standardized best-practice surgical techniques during implantation. Over the years trained veterinarians left projects, were promoted or otherwise lost to performing surgeries, such that surgical techniques diverged from the original standards and may have suffered in consequence. The reality of rehabilitation center working schedules and practices may also result in limited veterinary continuity, no guarantee of expert tuition or prior experience, and several different vets being required to carry out the procedure. It is important, however, to note that, although slightly different in application, intra-abdominal VHF implantation surgeries have also led to documented problems, including hemorrhage and infection among relocated river otters (42), and the rejection of a subcutaneous implant in a harbor seal almost a year after surgery (43).

Apart from a few documented cases where direct damage (e.g., bites, repeated self-inflicted blunt trauma, and heavy bumps or branch falls) was directly witnessed by project staff, most causes of faults are yet to be identified. Compounding the difficulty of diagnosing prematurely failed implants is the fact that relocating and capturing the animal in question, as well as removing the damaged implant can be a highly disruptive undertaking, especially considering the sensitivity of many rehabilitated apes to the adaptive process of reintroduction (16), and the inherent risks associated with even simple surgeries i.e., darting, anesthesia, and infection. The death of one orangutan during an implant removal surgery, while only an indirect result of IRT and its methodology, nonetheless supports this view. It must also be noted that an unknown number of devices may have failed and not be known to have failed within the large censored population reported here. These device failures may result in host animals never being relocated, while having to carry cracked implants for the rest of their natural life. The relative newness of this technology means that the true impact of leaving faulty implants within long-lived animals may never be known, so project leaders must decide if the risks presented here are balanced by the potential benefits of long-term post-release detection.

During the course of everyday orangutan locomotion and activity it is difficult to envisage a scenario where direct pressure is sufficiently exerted on the back of an individual's neck to result in a cracked casing. However, it may be that damage is caused, and certainly worsened, when animals are sleeping on their backs in nests, or when using their necks as a fulcrum for doing roly-polys, an occasional form of terrestrial locomotion used by some animals. The 18.1% total device failure rate within 1 year of activation reported here, while high, is nonetheless considerably lower than implanted equipment failures reported in Brown bears (44), although this is likely due to the considerably more complex procedure required to implant both the transmitter and an external antenna. Similarly, evidence from the deployment of new and emerging satellite technologies in a range of large mammals (45) suggests that researchers should keep in mind the very high risk of equipment failure; the same can be said, albeit to a lesser extent, for IRT.

While it is a key strength of IRT that all components are housed within one small unit, the cracked casings present in our study suggest an urgent need to investigate adaptations to make the implant housing more secure and inert. This was partly addressed several years ago when implant developers changed casing thickness from 0.8 to 12 mm after discovery of the first faulty batch of implants found with hairline cracks. Since that time, however, cracks have consistently been found in removed implants, so if casings cannot be made to survive intact within the body of an ape for 40–50 years without dramatically reducing signal transmission, then a new implant design must be considered to remove all risk of injury to focal animals. Assuming an animal is adequately monitored and survives until the end of an expected device lifespan, if it is then dying several years later, potentially with dependent offspring, due to unknown or as yet undocumented deleterious effects of its implant, this loss is magnified within the longer-term context of any reintroduction project. Minimizing the risk of anything that might jeopardize an individual's long-term survival is therefore vital on both welfare and conservation grounds. We note, however, that risk is by no means unique to the implantation method; all external radio telemetry attachments should be field tested to ensure the absence of deleterious effects (46). The reported death of at least two red howler monkeys from a screwworm larvae infestation that developed under their radio collars (47), and changes to the demographic integrity of newly collared owl monkeys returning to their social groups are evidence of this (13). It's worth also remembering that other damaging effects caused by radio telemetry applications may remain unreported (48).

### Conclusion and Recommendations

The importance of being able to regularly relocate reintroduced individuals is highlighted by the fact that both rehabilitated and wild translocated primates are most vulnerable immediately following release (24, 49). Radio telemetry therefore has a vital role to play in improving the long-term survival of individuals released. Implanted radio telemetry is directly responsible for the proliferation of scientific data collection on a widely reintroduced, yet Critically Endangered species, about which so little was previously known. With large sums of money channeled into great ape rehabilitation programmes worldwide, these data are now helping donors and conservationists to identify whether they are getting value in the strategies used, and outcomes produced, through reintroduction. Additionally, the generally positive perception of IRT among its practitioners demonstrates the clear and ongoing need for effective and reliable tracking methods for hard-to-monitor species like the orangutan.

Given the rapid pace of technological development and miniaturization of battery power sources in particular, implanted VHF transmitters may indeed be rendered superfluous within a few years. Notwithstanding, they currently remain the only viable, and robustly tested, option for the monitoring of orangutans. It is universally accepted that the current iteration of the implant must be improved to prevent faults and increase functionality. Most urgently, transmitter casings must be made more secure and shatter-proof. Its risks for some animals, namely splintered ceramics, assumed battery leakage, self-inflicted trauma and stress, and long-term post-operative recovery periods must, however, be balanced with the alternative of releasing animals with no monitoring device. If reintroduction is to serve its primary conservation function of re-establishing viable populations of threatened species, then all data on post-release outcomes and behavior are vital to promote survivorship. IRT is one such tool that has been developed to facilitate data collection. We hope that the results discussed here will lead to the improvement of this and other emerging technologies designed to facilitate the post release monitoring of hard-to-monitor species, not just those within the primate order.

## Ethics Statement

Procedures in Malaysia, Indonesia, Gabon and the Congo were carried out according to the requirements of national animal welfare and animal use legislation by registered and qualified veterinarians in registered institutions, IUCN Guidelines for Reintroductions and Other Conservation Translocations. No additional permits were required.

The main issue is that in most of the countries we work, there are no specific national animal welfare and animal use legislation beyond the permit to work on the animals and this is covered by the stringent permits of the registered institutions [Indonesia, Gabon, Congo] or government institutions [Malaysia].

## Author Contributions

JR and CW conceived and designed the study. GF, TP, MA, BG, and RS contributed to the conception and design of the study. JR wrote the manuscript and conducted statistical analysis. SH and NH wrote sections of the manuscript. JR, SH, AF, IS, MN, KL, AW, and PP acquired, categorized and provided data for the work. All authors contributed to manuscript revision, read and approved the submitted version.

### Conflict of Interest Statement

The authors declare that the research was conducted in the absence of any commercial or financial relationships that could be construed as a potential conflict of interest.

## Appendix

**Appendix A1:** Practitioner perceptions of the benefits and limitations of IRT and their recommendations for future development.

***Benefits of IRT***	***Limitations of IRT***	***Desired improvements/recommendations***
“*Couldn't identify that orangutans were adapting well/success rates only possible with this technology—many orangutans have disappeared and been re-found months later in different locations. Needed it for various interventions: provision of supplementary feeding; repatriation for medical treatment; prevention of crop-raiding. I wouldn't want to do reintroduction without telemetry for these latter reasons—early diagnosis of problems, and intervention, only possible for this many OU's with telemetry.”*		
“*Currently there is no better radio telemetry method for monitoring orangutans, and PRM without the use of radio telemetry has proved very difficult.”*		
“*Great advance in tracking technology” and “Observers who are monitoring released animals can find and identify individuals more efficiently.”*		
“*The scheduling capability of IRT means I can modify monitoring protocols and transmission schedules based on available resources and individual progress.”*		
“*It does make relocating an animal much easier, especially in the first several months post release before ranging habits have stabilized.”*		
“*The devices I implanted sub-cutaneously were a good size and caused no significant surgical problems but, of course, the smaller the better, i.e., easier to implant.”*		
“*It's easy to pass an orangutan that is right under your nose until they have established routines and ranging patterns, and then the result of the re-introduction remains unknown, and possibly the investment of over 5 years of rehab is lost (or at least is difficult to justify).”*	“*Topography and natural obstacles, e.g., dense vegetation, can distort and dampen the signal, leading to the short range of the radio signal and poor signal reception.”*	
“*If a device fails prematurely, it's unclear why it has stopped functioning. Given that a number of the devices have had cracked casings while still inside orangutans that have been released, a prematurely failed device may potentially pose a direct danger to an animal's well-being.”*		
“*Faulty receivers: 1) broken or weak signals from some units; 2) apparent interference between transmitters—the picking up of one signal when a different OU is nearby. Sometimes we are right in front of an orangutan and the receiver does not record their signal; even though the next day with a different receiver unit we pick it up.”*		
“*Skill and discipline in using the receivers, combined with tedious entangling of the antenna in the rain forest.” “Given that the implants are expensive and that a fair number of implants have already become broken or have failed, it is currently neither cost-effective nor time-effective to utilize the chips in all animals.”*	“*The incorporation of GPS/satellite technology would be a big help, so we could record where they had been traveling; and pick up signals from further distances. Would be nice to have positional data sent so can go direct to location to observe.” “Ideally, if small implants (like now) could transmit GPS signals so one could monitor their whereabouts from outside the forest.” “More powerful transmissions with a wider VHF signal range in mountain and dense forest.” “Is it possible to have stronger signals without interfering with wellbeing/health?” “Implants with increased battery lives and the ability to switch on and off remotely would be very useful, to preserve battery life and change the transmission schedule, according to logistics or the time of year.” “Better/more durable casings, longer/more efficient battery life, and greater signal range.” “Ensure that no device will fail.” “Smaller size to enable easier implanting with less tissue reaction.”*	
